# Preadipocyte Uncoupling Protein 1 Expression Invoked by Fibroblast Growth Factors Imprints on Post‐Differentiation White and Brown Adipocyte Phenotype

**DOI:** 10.1096/fj.202502663R

**Published:** 2025-10-16

**Authors:** Katharina Kuellmer, Carolin Mönch, Wilma Roch, Christine Wurmser, Patricia S. S. Petersen, Hildigunnur Hermannsdóttir, Mersiha Hasic, Brice Emanuelli, Tobias Fromme

**Affiliations:** ^1^ Chair of Molecular Nutritional Medicine, TUM School of Life Sciences Technical University of Munich Freising Germany; ^2^ Chair of Animal Physiology and Immunology, TUM School of Life Sciences Technical University of Munich Freising Germany; ^3^ Novo Nordisk Foundation Center for Basic Metabolic Research, Faculty of Health and Medical Sciences University of Copenhagen Copenhagen Denmark

**Keywords:** adipocyte differentiation, fibroblast growth factor signaling, inflammatory signaling, non‐thermogenic UCP1, preadipocyte programming, single‐cell RNA‐sequencing, uncoupling protein 1

## Abstract

Uncoupling protein 1 (UCP1) is a hallmark of thermogenic adipocytes and enables heat production by dissipating energy from mitochondrial proton motive force as heat. The purpose of its recently discovered presence in preadipocytes in response to certain fibroblast growth factors (FGFs) remains elusive. In this study, we systematically investigated the potential of all paracrine FGFs to invoke UCP1 expression in murine preadipocytes derived from interscapular brown and inguinal white adipose tissue. FGF2, FGF4, FGF8, and FGF9 induced UCP1 expression in undifferentiated preadipocytes, with FGF2 acting most potently and rapidly. This premature UCP1 induction did not translate into increased UCP1 thermogenic activity after complete adipogenic differentiation. Notably, preadipocyte treatment with FGFs and parallel UCP1 expression led to a sustained suppression of interferon‐stimulated genes after differentiation. Preadipocyte UCP1 was required and sufficient for this lasting imprint. Thus, FGF‐induced UCP1 expression in preadipocytes programs a lasting post‐differentiation anti‐inflammatory status.

## Graphical abstract

Primary preadipocytes from inguinal white or interscapular brown fat were treated with paracrine fibroblast growth factors (FGF) 2, 4, 8, or 9. Treatment induced acute Ucp1 expression in preadipocytes, independent of other browning markers. Upon subsequent differentiation, Ucp1 and browning markers were expressed at levels comparable to untreated cells. In contrast, inflammatory markers of fully differentiated adipocytes were strongly downregulated. These FGFs imprint a lasting, UCP1‐mediated effect in adipocytes.

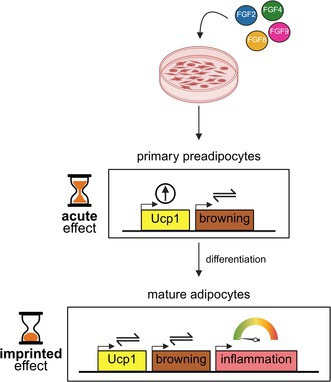

## 1 Introduction

Uncoupling protein 1 (UCP1) is a protein of the mitochondrial inner membrane and a central mechanistic component of non‐shivering thermogenesis [1]. Activated by free fatty acids, it uncouples oxygen consumption from ATP synthesis by mediating a proton flux from the intermembrane space into the mitochondrial matrix. The expression of UCP1 is thought to be strictly limited to thermogenic brown or beige adipocytes [2]. The former are found within the specialized mammalian heater organ brown adipose tissue (BAT), and the latter are interspersed within otherwise white adipose tissue depots [3]. The recruitment and differentiation of these thermogenic adipocyte populations are of considerable importance, as the efficacy of potential drugs targeting non‐shivering thermogenesis to combat metabolic disease is constrained by the limited total amount of brown and beige fat—typically only around 300 g in a healthy adult human [4, 5].

We and others previously reported the surprising discovery of UCP1 in non‐differentiated preadipocytes in response to treatment with fibroblast growth factors (FGF) 6, 8, and 9 [6, 7, 8]. These FGFs are members of an ancestral subgroup of FGFs featuring a heparin anchor, rendering their mode of action to be paracrine signaling as opposed to the widely known endocrine FGFs, for example, FGF21 [9, 10, 11]. Paracrine FGFs bind FGF receptors (FGFRs) with heparin as a cofactor instead of the obligatory Klotho co‐receptors of endocrine FGFs [12, 13, 14]. They signal through one or several of the four FGF receptors with FGF‐specific affinity patterns (FGFR1‐4), with two major isoforms in immunoglobulin‐like domain III (IIIb and IIIc) that influence binding specificity and affinity [15, 16]. UCP1 expression is induced in preadipocytes by FGF8 through a signaling cascade emanating from FGFR1 and requires both prostaglandin synthesis and a high glycolytic flux [6].

In this study, we screened all paracrine‐acting FGFs for their potential to induce UCP1 expression in preadipocytes, comprehensively described the resulting cell type, and identified a programming effect of FGFs that is maintained throughout cell differentiation to fine‐tune the inflammatory status of mature adipocytes.

## 2 Materials and Methods

### 2.1 Mice

All mice were maintained at 23°C ambient temperature and 55% relative humidity, with a 12 h light/dark cycle in our specified‐pathogen‐free mouse facility. Mice had ad libitum access to a standard maintenance and breeding chow diet (Ssniff Spezialdiäten GmbH, Germany) and water. Paracrine FGF screening was performed with primary preadipocytes isolated from the UCP1 dual‐reporter gene mouse C57BL/6NTac‐UCP1tm3588, simultaneously expressing firefly luciferase and near‐infrared fluorescent protein 713 [17]. Subsequent studies were conducted in primary preadipocytes derived from Bl6N wildtype mice as indicated.

### 2.2 Fibroblast Growth Factors

Recombinant paracrine fibroblast growth factors were purchased from R&D Biosciences, specifically mouse FGF1 (4686‐FA‐025/CF), FGF2 (3139‐FB‐025/CF), FGF4 (7486‐F4‐025/CF), FGF6 (5750‐F6‐025/CF), FGF7 (5028‐KG‐025/CF), FGF8b (423‐F8‐025/CF), FGF9 (7399‐F9‐025/CF), FGF10 (6224‐FG‐025/CF), FGF17 (7400‐FG‐025/CF); and recombinant human FGF3 (1206‐F3‐025/CF), FGF5 (237‐F5‐050/CF), FGF16 (1212‐FG‐025/CF), FGF18 (8988‐F18‐050), FGF20 (2547‐FG‐025/CF), FGF22 (3867‐FG‐025/CF). All lyophilized FGFs were reconstituted following the manufacturer's instructions in phosphate buffered saline or water to a stock concentration of 3–24 μM and diluted to the indicated target concentration in appropriate cell culture medium. For FGF screening, primary preadipocytes were treated with 5.5 nM FGFs (1–10, 16–18, 20, and 22). Dose–response experiments were performed for FGF2, FGF4, FGF8, and FGF9 using two‐fold serial dilutions from 100 nM down to 0.2 nM (10 steps). Cells were treated with these dilutions for 48 h before detection of luciferase activity; see below. For time‐course experiments, cells were treated with 5.5 nM FGF for the indicated durations (0–72 h) before washing and lysis.

### 2.3 Cell Culture

Primary preadipocytes isolated from brown and inguinal white adipose tissue were immortalized by retroviral transduction with SV40 large T antigen using pBABE‐puro SV40 LT and selected with puromycin, as previously described [6]. Immortalized cells were cultured and differentiated in high‐glucose Dulbecco's Modified Eagle Medium (4.5 g/L glucose, Sigma‐Aldrich) with 10% Fetal Bovine Serum (FBS Supreme, PAN Biotech). For isolation of primary cells, the Stromal Vascular Fraction (SVF) was isolated from interscapular brown or white adipose tissue of 8–10 week‐old C57BL/6N mice, minced until homogenous, and digested using collagenase (Cellsystems). Digested tissues were filtered through a 250 μM nylon mesh and centrifuged at 250 g for 5 min. The resulting SVF pellet was resuspended in wash buffer (1× HBSS w/Mg; Ca + 3.5% Bovine Serum Albumin) and centrifuged at 500 g for 5 min. Isolated primary cells were hereon cultured in 20% FBS‐supplemented high‐glucose DMEM until 80% confluency and treated or differentiated in 10% FBS‐supplemented high‐glucose DMEM.

For treatment of both primary and immortalized preadipocytes, paracrine FGF recombinant proteins were added to the culture media, with 5.5 nM used as the standard working concentration unless otherwise specified (carrier‐free, R&D Systems). Subsequent differentiation was induced with an induction cocktail consisting of maintenance media with added 850 nM insulin, 1 nM T3, 500 μM IBMX, 1 μM dexamethasone, 125 μM indomethacin, and 1 μM rosiglitazone. After 48 h, the induction medium was replaced with 850 nM insulin, 1 nM T3, and 1 μM rosiglitazone. Cells were differentiated for 6 days. FGFR inhibitor (LY2874455, MCE) was used simultaneously at indicated concentrations throughout FGF treatment.

### 2.4 Transfection of WT‐1‐CRISPRa‐SAM Preadipocytes

The WT‐1‐CRISPRa‐SAM cell line is designed to activate the expression of target genes through stable expression of CRISPRa (dCas9‐VP64) and Synergistic Activation Mediator (SAM) complex [18]. sgRNAs targeting either UCP1 or FGF2 were subcloned into the sgRNA (MS2) cloning backbone (Addgene, #61424) using a Golden Gate reaction as previously described [18, 19]. For transfection, the WT‐1‐CRISPRa‐SAM cells were seeded onto 24‐well plates and transfected upon reaching confluency by mixing 250 ng of sgRNA with TransIT‐X2 (Mirus, #MIR6000) transfection reagent according to the manufacturer's instructions. Forty‐eight hours post‐transfection, the cell culture medium was replaced, and after an additional 48 h, the transfected preadipocytes were harvested or differentiated for subsequent analyses. sgRNA sequences are listed in Table 1.

**TABLE 1:** sgRNA sequences for CRISPRa.

sgRNA	Guide sequence
*Fgf2*	CGTAGAGCACAAGCTGTGTAAGG
*Ucp1*	GGGAGTGACGCGCGG CTGGG

### 2.5 Bioluminescence Assay

Luciferase activity was quantified as an indirect measure of UCP1 expression (UCP1‐LUC) in cell lysates derived from the UCP1‐reporter mouse model. Luminescence was measured with the InfiniteM2000 NanoQuant (Tecan) using the luciferase assay system kit (E1501, Promega). Briefly, cells were washed with phosphate‐buffered saline (PBS) once and lysed in cell culture lysis reagent, shaking for 30 min at 20 rpm. A total of 10 μL of cell lysate was added to 50 μL of luciferase assay buffer on a white 96‐well plate. Luciferase assay buffer was added, and luminescence was measured over an integration time of 10 ms directly after. Luminescence was normalized to non‐treated and FGF1‐treated cells.

### 2.6 Respirometry

Immortalized brown preadipocytes were treated with paracrine FGFs (1, 2, 4, 8, and 9). Oxygen consumption rate (OCR) was either directly measured or cells underwent differentiation as stated above. Oxygen consumption rate (OCR) was either directly measured or cells underwent differentiation as stated above, following OCR measurement [20]. Preadipocytes were measured in mitochondrial respiration medium (MiR05), prepared according to the formulation provided by OROBOROS Instruments (Innsbruck, Austria), and contained 0.5 mM EGTA, 3 mM MgCl_2_, 60 mM lactobionic acid, 20 mM taurine, 10 mM KH_2_PO_4_, 20 mM HEPES, 110 mM sucrose, and 1 g/L BSA (bovine serum albumin, essentially fatty acid‐free), and was adjusted to pH 7.1. Fully differentiated adipocytes were measured in Seahorse medium, prepared using a DMEM base (Sigma D5030, 8.3 g/L) supplemented with 25 mM glucose, 2 mM GlutaMAX, 2 mM sodium pyruvate, 31 mM NaCl, 25 mM HEPES, and 2% (w/v) essentially fatty‐acid‐free BSA. A Seahorse XF Pro Sensor Cartridge (Agilent Technologies) was hydrated overnight with XF calibrant in a CO_2_‐free incubator at 37°C. For measurements, cells were washed twice with the appropriate assay medium, and 180 μL of assay medium was added to each well. The washed cells were incubated at 37°C for 1 h in CO_2_‐free conditions using the Cytation 1 imaging device (BioTek Cytation 1 Cell Imaging Multimode Reader, Agilent Technologies) to ensure proper outgassing. Cell Imaging software (Agilent Technologies) captured bright‐field images of the cells automatically during incubation. To measure UCP1‐dependent respiration in preadipocytes, cells were permeabilized by 20 μM α‐chaconine (Extrasynthese 1553 S) [21]. Following cell lysing, 20 μM 4‐[(1E)‐2‐(5,6,7,8‐tetrahydro‐5,5,8,8‐tetramethyl‐2‐naphthalenyl)‐1‐propen‐1‐yl]‐benzoic acid (TTNPB, T3757 Sigma‐Aldrich) was injected for direct UCP1 activation. A total of 1 μM carbonyl cyanide p‐trifluoromethoxy‐phenylhydrazone (FCCP) and 5 μM Antimycin A were injected afterwards. UCP1‐dependent respiration in adipocytes was measured with the following injection scheme: 5 μM Oligomycin, 0.5 μM Isoproterenol, 1 μM FCCP, 5 μM Antimycin A. Oxygen consumption rate (OCR) was recorded in response to the experimental substrates. Data visualization and initial analysis were performed using Wave Pro software (Agilent Technologies).

### 2.7 Oil Red O Staining

Oil Red O working solution was prepared by mixing three parts of Oil Red O stock solution (0.5% w/v Oil Red O, 100% v/v Isopropanol, filtered through a 0.45 μM filter) with two parts of double‐deionized water (ddH_2_O), followed by filtration through a 0.22 μm filter (Berrytec). Fully differentiated cells were washed twice with pre‐warmed PBS and fixed with 100 μL/well of 3.7% (v/v) formaldehyde for 1 h at room temperature. Fixed cells were washed three times with ddH_2_O and once with 60% isopropanol for 5 min, then air‐dried. Cells were incubated for 15 min in a freshly prepared Oil Red O working solution. Excess stain was removed by washing four times with ddH_2_O. Stained lipid droplets were visualized under a light microscope at 20× magnification (Axiovert 40 CFL, Carl Zeiss). For quantification and size distribution analysis, images of unstained cells were analyzed using the WimLipid pattern recognition algorithm (Wimasis, Onimagin Technologies).

### 2.8 Quantitative RT‐PCR

Cells were harvested and lysed with TRIsure reagent, followed by RNA extraction using the SV Total RNA Isolation System (Promega). The RNA concentrations were measured using a Nanodrop‐1000 instrument. cDNA was synthesized from 500 to 1000 ng RNA using the SensiFast cDNA Synthesis Kit (Bioline). RT‐qPCR analysis was performed using the LightCycler480 Real‐Time PCR System (Roche) with the SensiMix SYBR No‐ROX Kit (Bioline). The relative standard curve method was employed for quantification using pooled sample cDNA standards. Target gene expression was normalized to the reference genes *Tbp, Hsp90*, and *Tf2b*. Primer sequences are listed in Table 2.

**TABLE 2:** forward and reverse primer sequences.

Gene	5′–3′ forward primer	5′–3′ reverse primer
*Ifnb1*	TCCAGCTCCAAGAAAGGACG	TTGAAGTCCGCCCTGTAGGT
*Ifnar2*	ACACCCTCTGGTACACAGTCA	AAGTGGCGGAAGTTCAAGACA
*Irf7*	AAACCATAGAGGCACCCAAG	CCCAATAGCCAGTCTCCAAA
*Il6*	TGGTACTCCAGAAGACCAGAGG	AACGATGATGCACTTGCAGA
*Fgf2*	GAAACACTCTTCTGTAACACACTT	GTCAAACTACAACTCCAAGCAG
*Ucp1*	TCTCTGCCAGGACAGTACCC	AGAAGCCCAATGATGTTCAG
*Tbp*	ACCCTTCACCAATGACTCCTATG	TGACTGCAGCAAATCGCTTGG
*Hsp90*	CCACCACCCTGCTGCTCTGTACTA	GGGACATGAGCTGGGCAATTT
*Tf2b*	TGGAGATTTGTCCACCATGA	GAATTGCCAAACTCATCAAAACT

### 2.9 bulkRNA‐Sequencing

Primary or immortalized preadipocytes were treated with vehicle or FGF1, FGF2, FGF4, FGF8, or FGF9 for 48 h. FGF‐treated cells were either directly harvested or differentiated as stated above and harvested at different time points as indicated. Harvested cells were lyzed in TRIsure and RNA was extracted using the SV Total RNA Isolation System by Promega following the manufacturer's protocol. RNA quality and integrity were assessed using Agilent Bioanalyzer 2100. High‐quality RNA (RIN > 7) was used to prepare libraries using the Illumina stranded mRNA Kit, the Illumina stranded mRNA Kit, and the UDI kit. Sequencing was performed on Illumina NovaSeq 6000, generating paired‐end reads of 150 bp. Raw reads were trimmed for quality using TrimGalore and aligned to the reference genome, GRCm39, using HISAT2. Gene‐level counts were generated with featureCounts and compiled into a count matrix for subsequent analysis. Count data were analyzed using DESeq2 (v1.42.1). A variance‐stabilizing transformation (VST) was applied to normalize the data. PCA and heatmaps were visualized with ggplot2 (v. 3.5.2) and pheatmap. All analyses were conducted in R (v. 4.3.3).

### 2.10 Single‐Cell RNA‐Sequencing

Primary preadipocytes isolated from iBAT and iWAT were cultured until 80% confluency and subsequently treated with vehicle, FGF1, or FGF2 as stated above for 48 h. Cell suspensions were loaded into the BD Scanner to count cells and assess cell viability by brightfield and dual‐band fluorescence imaging. Samples were tagged using Sample Tags (BD Single‐Cell Multiplexing Kit, Anti‐Mouse MHC‐H2 Class I, clone M1/42) and merged. In total, 40 000 cells were loaded into the BD Rhapsody Cartridge using the BD Rhapsody Xpress system. Reverse transcription and cDNA amplification were performed with the BD Rhapsody cDNA kit as stated by the supplier (protocol 23‐22 951(02)). Libraries were prepared using the BD Rhapsody Whole Transcriptome Amplification kit (protocol 23‐24 119(02)). Samples were sequenced on a NovaSeq X Plus platform in a paired‐end run (PE150), Illumina by Novogene. Reads were aligned and annotated using the data pipeline at Seven Bridges Genomics. Subsequent analysis was performed using Seurat (v. 5.0.0). Mitochondrial gene expression percentages were calculated for each cell to assess overall cell quality. Cells were filtered based on the number of detected features per cell (nFeature_RNA > 200 and < 7500) and the percentage of mitochondrial transcripts (< 30%). Normalization of the dataset was performed using log‐normalization to adjust for variability in sequencing depth. To account for the confounding effects of cell‐cycle variation, cell‐cycle phase scores were computed using previously published gene markers [22]. A difference score was calculated (S.Score—G2M.Score) and regressed during data scaling. Dimensionality reduction was performed using principal component analysis (PCA), and the optimal number of dimensions for clustering was determined by Elbow Plot. Clustering was conducted using shared nearest neighbor (SNN) graphs, and the clusters were visualized using uniform manifold approximation and projection (UMAP). Clusters were annotated to cell subtypes by known marker genes based on the marker genes for each cluster. Known cell type marker genes were collected from CellMarker2.0 [23].

### 2.11 Statistical Analysis

Statistical analyses were conducted using GraphPad Prism version 10.4.2 (GraphPad Software, San Diego, California, USA). Parametric tests, including Student's *t*‐test and one‐way or two‐way ANOVA with Bonferroni correction, Dunnett's or Tukey's multiple comparisons test, were applied to data that followed an approximately normal distribution. Data are reported as means ± SD unless specified otherwise in the figure legends. Statistical significance was set at *p* < 0.05 and denoted with asterisks: *p < 0.05.

## 3 Results

### 3.1 Paracrine Fibroblast Growth Factors 2, 4, 8, and 9 Induce UCP1 Expression in Primary White and Brown Preadipocytes

**FIGURE 1:**
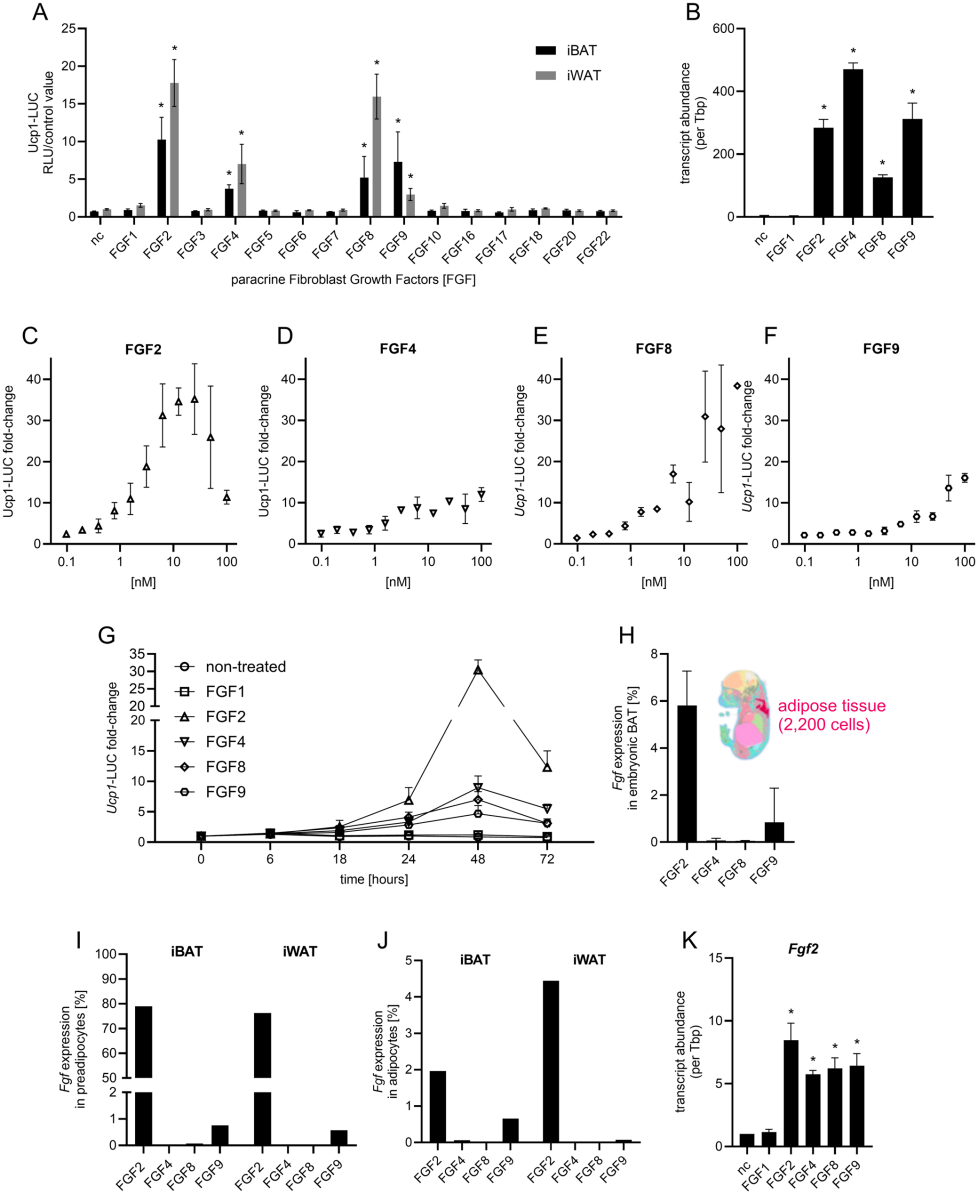
Paracrine fibroblast growth factors 2, 4, 8, and 9 induce Ucp1 expression in brown and white preadipocytes. (A) Luciferase UCP1 reporter activity upon treatment with individual FGFs in brown and white preadipocytes from *Ucp1*‐ reporter mice. (B) *Ucp1* mRNA abundance normalized to *Tbp* in immortalized brown preadipocytes treated with 5.5 nM FGF1, FGF2, FGF4, FGF8, or FGF9 for 48 h, compared to untreated controls. (C–F) Dose response curves of *Ucp1* reporter luminescence in white preadipocytes treated with increasing concentrations of FGF1, FGF2, FGF4, FGF8, or FGF9 (maximum 100 nM, 10 serial two‐fold dilutions). (G) Time‐course of *Ucp1*‐linked luminescence in white preadipocytes treated with 5.5 nM FGF1, FGF2, FGF4, FGF8, or FGF9. (H) Expression of FGF2, FGF4, FGF8, and FGF9 in annotated embryonic brown adipose tissue (E16.5) extracted from the spatial transcriptome database MOSTA (website: https://db.cngb.org/stomics/mosta/, Chen et al., 2022). (I, J) Expression of FGF2, FGF4, FGF8, and FGF9 in pre‐ I or mature J adipocytes from adult iWAT and iBAT, based on single‐cell RNA‐seq dataset. Data represent one dataset per tissue. (K) *Fgf2* mRNA abundance normalized to *Tbp* in immortalized brown preadipocytes treated with 5.5 nM FGF1, FGF2, FGF4, FGF8, or FGF9 for 48 h, compared to untreated controls. In all panels data represent mean ± SD; *n* = 3, unless otherwise stated. Statistical analysis: One‐ (B, H, K) or two‐way (A, C) ANOVA with Dunnett's (A–C, K) or Tukey's (H) multiple comparisons test. *p < 0.05.

Fibroblast growth factor 8 (FGF8) induces the atypical expression of uncoupling protein 1 (UCP1) in white and brown preadipocytes via FGF receptor 1 (FGFR1) [6]. The FGFR1 is a promiscuous receptor interacting with several FGFs with varying affinity [9]. We conducted a comprehensive screening using primary preadipocytes derived from a luciferase‐based UCP1 reporter mouse to explore the potential of all paracrine‐acting fibroblast growth factors (FGFs 1–10, 16–18, 20, and 22) to drive UCP1 expression. The model features a luciferase open reading frame inserted into the endogenous UCP1 locus, enabling indirect measurement of UCP1 gene transcriptional activity via luminescence [17]. Primary preadipocytes from interscapular brown adipose tissue (iBAT) and inguinal white adipose tissue (iWAT) were harvested and treated with each FGFs, respectively. The factors FGF2, 4, 8, and 9 proved to be potent inducers of UCP1 expression across primary preadipocytes derived from both iBAT and iWAT, with FGF2 eliciting the strongest response, followed by FGF8, FGF9, and FGF4 (Figure 1A,B). In dose–response experiments, FGF2 and 8 induced UCP1 reporter activity at lowest (nanomolar) concentrations (Figure 1C–F). FGF 4 and 9 required higher concentrations to initiate UCP1 reporter activity, and no plateau was yet reached at the maximum tested concentration of 100 nM. We assessed the efficacy of these FGFs over 3 days, including shorter intervals of 6 and 18 h to capture potential rapid, transient effects. UCP1 expression increased steadily, peaking at 48 h, with no earlier transient surge (Figure 1G).

FGFs are recognized primarily for their roles in embryonic development, particularly in brain, limb, and central nervous system formation [11]. Both white and brown adipose tissue depots develop late during embryogenesis [24], and FGFs may play a physiological role during these events. We thus explored the presence of UCP1‐inducing FGFs 2, 4, 8, and 9 in both mature and developing adipose tissues. We re‐analyzed the publicly available Mouse Organogenesis Spatiotemporal Transcriptomic Atlas (MOSTA, [25]) as well as our own datasets, that is, single‐cell RNA‐sequencing (scRNA‐seq) datasets from primary brown and white preadipocytes, and single‐nuclei RNA‐sequencing (snRNAseq) datasets from mature brown and white adipocytes.

In developing adipose tissue, FGF2 displayed the highest expression compared to FGF4, FGF8, and FGF9 at embryogenic stage E16.5 (Figure 1H). In primary preadipocytes from both inguinal white and interscapular brown adipose tissue, only FGF2 was consistently expressed, while FGF4, FGF8, and FGF9 were absent in preadipocyte populations (Figure 1I). Similarly, FGF2 was the only present FGF in mature adipocytes derived from iWAT and iBAT (Figure 1J). Taken together, these findings point towards FGF2 as a relevant driver of UCP1 expression in vivo. Interestingly, treatment with FGF4, FGF8, or FGF9 (but not control FGF1) consistently increased FGF2 expression in preadipocytes, supporting FGF2 as the primary mediator of FGF‐induced UCP1 expression (Figure 1K).

In summary, FGFs 2, 4, 8, and 9 act as inducers of significant UCP1 transcript expression in both inguinal white and interscapular brown preadipocytes. Of these, FGF2 is predominantly expressed at all developmental stages of adipose tissue in mice as well as in mature adipose tissue preadipocytes and adipocytes.

### 3.2 Fibroblast Growth Factors 2, 4, 8, and 9 Signal via the Same Pathway to Induce UCP1 but Do Not Induce a ‘Browning’ Signature

The FGFR1 is essential for the induction of UCP1 expression by FGF8 [6]. The binding affinities of FGF2, 4, 8, and 9 to the group of FGF receptors are well in line with a common signaling path via FGFR1 [11]. FGF2 binds most strongly to the receptor isoforms FGFR1b and FGFR1c; FGF4 primarily binds FGFR1c and FGFR2c; FGF8 has the highest affinity for FGFR1c; and FGF9 binds most strongly to FGFR1c. Thus, FGFR1 isoforms are the likely receptors mediating the FGF–UCP1 axis in preadipocytes for all identified FGFs, even in the case that FGFs 4, 8, and 9 would not act via induction of the primary active agent FGF2.

**FIGURE 2:**
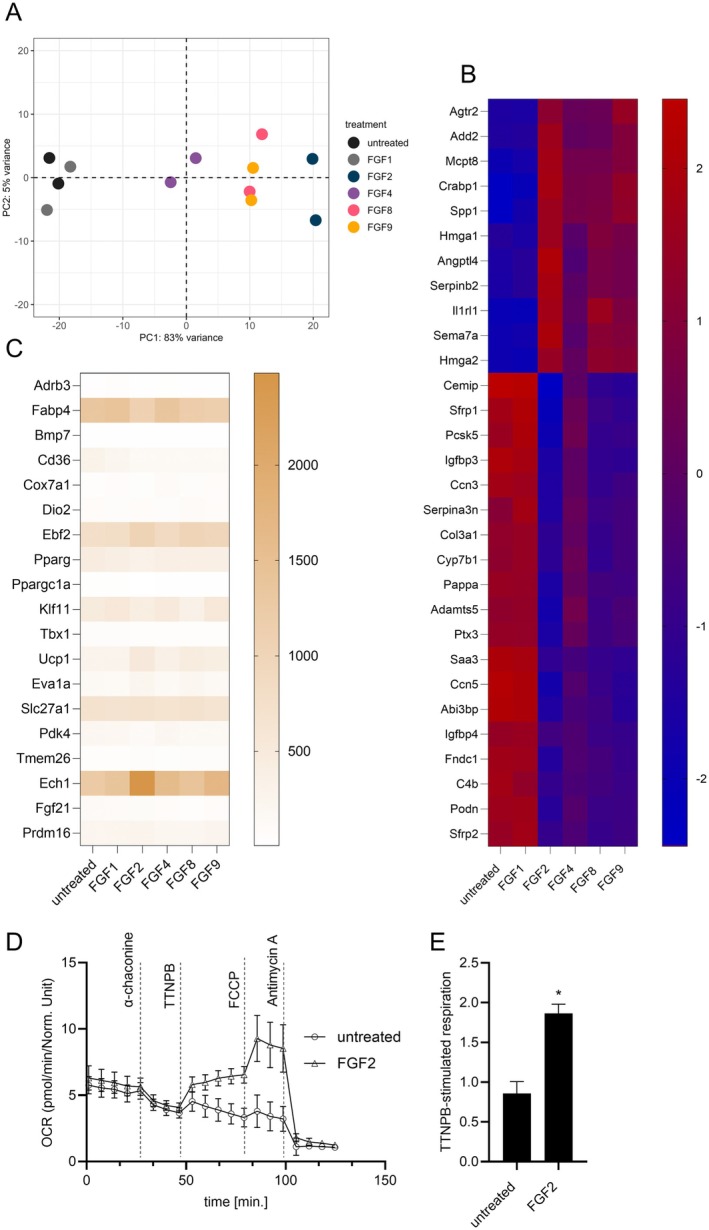
FGF2, FGF4, FGF8, and FGF9 induce a shared transcriptomic signature in white preadipocytes. (A) Principal component analysis (PCA) of global transcriptomic profiles from white preadipocytes treated with FGF1, FGF2, FGF4, FGF8, or FGF9 (5.5 nM, 48 h), compared to untreated controls. Each condition includes *N* = 2 biological replicates. PCA was performed using variance‐stabilized transformed counts. (B) Heatmap showing the top 20 most variable genes across all samples, selected based on variance‐stabilized expression values. Data are clustered by gene and sample to illustrate global transcriptional differences between treatments. (C) Heatmap displaying the expression of 25 selected browning‐associated genes in white preadipocytes treated with 5.5 nM FGF2, FGF4, FGF8, or FGF9 for 48 h, compared to untreated controls. Gene expression is based on normalized counts from bulk RNA‐seq data. Rows represent genes, and columns represent treatment groups. (D) Time course of oxygen consumption rate in immortalized brown preadipocytes comparing FGF2 treatment with its untreated control, measured by microplate‐based respirometry (Seahorse XF96 Analyzer). Oxygen consumption is recorded in lyzed cells under basal conditions and in response to successive injection TTNPB (15 μM), FCCP (1 μM), and antimycin A (5 μM) to determin direct UCP1 activity, maximal and non‐mitochondrial respiration, respectively. Mean values ± SD, *n* = 3 (biological replicates). (E) Quantification of TTNPB‐stimulated UCP1‐mediated uncoupled respiration, expressed as fold of basal leak respiration. Mean values ± SD, *n* = 3 (biological replicates). Statistical analysis: Unpaired *t*‐test. *p < 0.05.

We characterized the transcriptional response to differential FGF treatment in inguinal white primary preadipocytes by RNA sequencing. Principal Component Analysis (PCA) of FGF‐treated cells revealed a distinct effect of treatment compared to non‐treated and FGF1‐treated cells (Figure 2A). Clustering along the first principal component reflected the strength of UCP1 induction across FGFs, with FGF2 as the strongest inducer and FGF4 the weakest. FGFs 2, 4, 8, and 9 did not form separate clusters along any other principal component, corroborating a shared receptor and signaling pathway. Similarly, in a heatmap analysis of the most FGF‐sensitive transcripts, non‐treated and FGF1‐treated cells displayed a similar gene expression profile, distinct from a shared pattern induced by FGF2, FGF4, FGF8, and FGF9 (Figure 2B). Among these, FGF2‐, FGF8‐, and FGF9‐treated cells showed closely similar transcriptomic profiles, whereas FGF4 induced comparatively weaker changes—consistent with the separation observed in the PCA plot (Figure 2A). Surprisingly, the induction of the hallmark brown/beige adipocyte marker UCP1 was not part of an accompanying generalized browning phenomenon but occurred separately from the well‐characterized thermogenic expression signature (Figure 2C).

The absence of a typical environment that would enable thermogenic UCP1 activity in differentiated brown adipocytes prompted us to determine the functionality of FGF‐mediated UCP1 induction. Since preadipocytes lack beta‐adrenergic receptors and relevant intracellular lipid stores—both required to activate UCP1‐mediated mitochondrial uncoupling—we utilized a direct activator of UCP1 activity, the retinoic acid analog 4‐[(1E)‐2‐(5,6,7,8‐tetrahydro‐5,5,8,8‐tetramethyl‐2‐naphthalenyl)‐1‐propen‐1‐yl]‐benzoic acid (TTNPB) [26]. Remarkably, UCP1 in FGF2‐treated preadipocytes could indeed be activated to a considerable portion of maximal oxidative capacity, demonstrating proper localization to the inner mitochondrial membrane and principal functionality (Figure 2D,E). Additionally, this confirms UCP1 protein translation from the UCP1 mRNA transcript—indicating effective promoter transactivation and protein expression in preadipocytes in this study.

In summary, all four paracrine FGFs 2, 4, 8, and 9 induced the same transcriptomic signature in primary white preadipocytes, indicating a common receptor and signaling pathway. They do not initiate browning or differentiation per se. Importantly, UCP1 protein derived from FGF2 treatment was functional, suggesting an alternative role in this cell type different from *bona fide* thermogenesis.

### 3.3 Single‐Cell Analysis of FGF2‐Driven Preadipocyte Transition Reveals Persisting Preadipocyte Identity of UCP1 Expressing Cells

**FIGURE 3:**
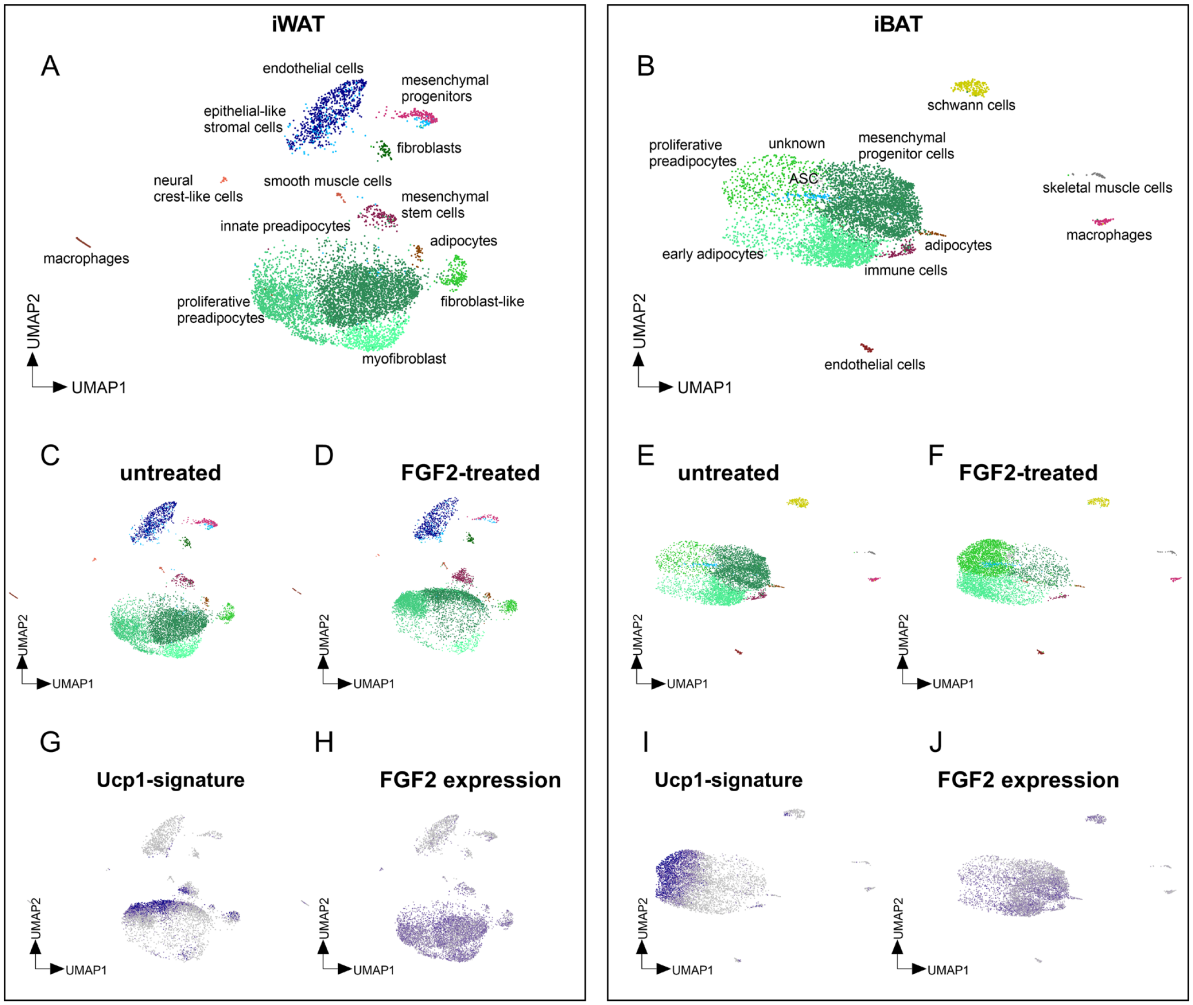
FGF2 treatment reshapes the cellular landscape of primary iWAT and iBAT preadipocytes. (A) UMAP of iWAT‐derived cells (stromal‐vascular fraction), revealing 12 distinct clusters, separated by colors. In the untreated condition, preadipocytes constitute the predominant population. (B) UMAP of iBAT‐derived cells(stromal‐vascular fraction), showing 11 distinct clusters, separated by colors. Similar to iWAT, untreated samples are primarily composed of preadipocytes. (C) UMAP of untreated iWAT preadipocytes showing the baseline distribution of clusters, and (D) UMAP of FGF2‐treated iWAT preadipocytes reveals a shift in cluster composition within the preadipocyte population. (E) UMAP of untreated iBAT preadipocytes showing the baseline cluster distribution and (F) UMAP of FGF2‐treated iBAT preadipocytes displaying a similar shift in cluster localization within the preadipocyte compartment. (G) Expression of a Ucp1 gene signature (top 20 upregulated genes from bulk RNA‐seq) in FGF2‐treated iWAT preadipocytes, specifically localized to the shifted preadipocyte population, and (H) FeaturePlot showing endogenous *Fgf2* expression in untreated iWAT preadipocytes. Cells expressing *Fgf2* are highlighted in blue; non‐expressing cells are shown in gray with *Fgf2* expression restricted to the subset of cells previously shown to respond with a *Ucp1*‐associated transcriptional shift. (I) Expression of the Ucp1 gene signature in FGF2‐treated iBAT preadipocytes, marking the same preadipocyte subpopulation that shifts upon treatment. (J) FeaturePlot showing endogenous *Fgf2* expression in untreated iBAT preadipocytes.

The cellular identity and biological role of FGF‐induced UCP1‐expressing cells is unclear. We performed single‐cell RNA sequencing (scRNA‐seq) to analyze which primary cell population is FGF2‐responsive and if such treatment induces a new and discrete UCP1‐positive subpopulation. Uniform Manifold Approximation and Projection (UMAP) dimensionality reduction of primary cells isolated and cultured from white and brown adipose tissue revealed 12 distinct clusters in iWAT and 11 clusters in iBAT, representing transcriptionally distinct cell populations (Figure 3A,B). These were manually annotated by marker genes derived from the CellMarker 2.0 database [23]. Three prominent clusters corresponding to preadipocytes and fibroblast‐like cell populations were identified along with smaller clusters representing immune and endothelial cells. In iWAT‐derived cells, FGF2 treatment caused a shift in subpopulations within the pre‐existing transcriptional space of identified preadipocytes (Figure 3C,D). Similarly, in iBAT‐derived cells, FGF2 treatment induced a pronounced shift in cell distribution within preadipocyte clusters (Figure 3E,F). Importantly, no entirely new subpopulation of preadipocytes or any other distinct cell type emerged in response to FGF2 treatment (Figure 3D,F). Thus, neither in primary cells from iWAT nor iBAT FGF2 re‐programmed preadipocytes into an entirely new subpopulation but rather altered the distribution into pre‐existing preadipocyte sub‐populations.

We verified the FGF2‐induced shift in preadipocyte characteristics to be associated with the initially observed induction of UCP1 expression. The top 20 transcripts correlated to UCP1 expression in the previous bulk RNA sequencing experiment (Figure 2B) mapped very well onto the preadipocyte population arising in response to FGF2 treatment (Figure 3G,I). This UCP1‐associated signature was observed in primary preadipocytes from both iBAT and iWAT, demonstrating that both respond to FGF2 by positively regulating UCP1 without forming a new, non‐preadipocyte subpopulation.

The signal FGF2 itself was already expressed by cell populations within the untreated primary cells, indicating ongoing FGF2 crosstalk in the absence of any manipulation (Figure 3H,J). Interestingly, these FGF2 positive cells themselves were nearly exclusively of the preadipocyte group, indicating a paracrine and/or autocrine path of communication.

Pathway analyses of FGF2‐responding preadipocytes demonstrated a significant upregulation in cell proliferation pathways, consistent with prior findings of FGF8‐mediated effects [6]. Pathways related to browning or adipogenesis were not enriched, in line with our earlier interpretation of global transcriptome changes. UCP1 expression in preadipocytes is independent of these well‐known differentiation processes and thus represents a distinct phenomenon within the preadipocyte lineage.

In summary, single‐cell RNA sequencing of iWAT and iBAT preadipocytes identified a distinct, naturally occurring preadipocyte population responsive to FGF2 treatment. Responding preadipocytes remained within the bounds of pre‐existing preadipocyte clusters rather than forming a new population. FGF2 itself also localized to preadipocytes, suggesting an autocrine and/or paracrine signaling path within the FGF2–UCP1 axis. Pathway analyses confirmed the absence of browning or adipogenesis induction, highlighting a unique FGF2‐driven UCP1 expression in preadipocytes while preserving their overall cellular identity.

### 3.4 Fibroblast Growth Factor‐Pretreated Adipocytes Differentiate Into Fully Functional Adipocytes

**FIGURE 4:**
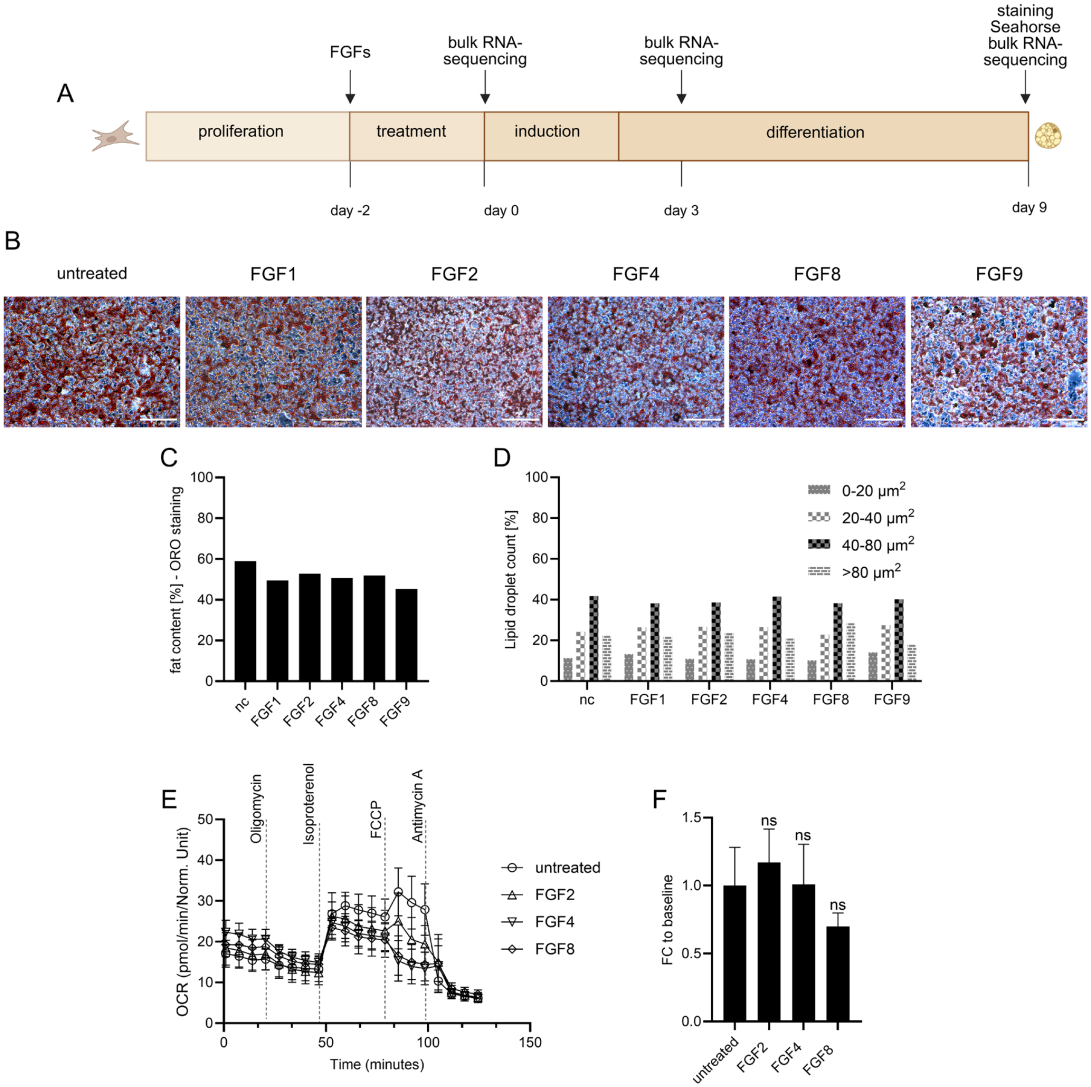
FGF pretreatment does not alter adipocyte lipid accumulation or droplet morphology when differentiated into adipocytes. (A) Experimental design showing preadipocyte treatment with FGF1, FGF2, FGF4, FGF8, or FGF9 followed by differentiation. (B) Oil Red O staining of lipid droplets in differentiated adipocytes pretreated with individual FGFs, compared to untreated controls (scalebar 50 μm). (C) Quantification of lipid content as a percentage of total well area stained, showing no significant differences across treatments. (D) Lipid droplet count per treatment group, stratified by droplet size (0–20 μm^2^, 20–40 μm^2^, 40–80 μm^2^, > 80 μm^2^), revealing similar distributions across all conditions. (E) Time course of oxygen consumption rate in immortalized brown adipocytes pretreated with FGF2, 4, or 8, compared with its untreated control, measured by microplate‐based respirometry (Seahorse XF96 Analyzer). Oxygen consumption is recorded under basal conditions and in response to successive injection of oligomycin (5 μM), isoproterenol (1 μM), FCCP (1 μM), and antimycin A (5 μM) to determine basal leak, UCP1‐dependent uncoupled, maximal, and non‐mitochondrial respiration, respectively. Mean values ± SD, *n* = 3 (biological replicates). (F) Quantification of isoproterenol‐stimulated UCP1‐mediated uncoupled respiration, expressed as fold of basal leak respiration. Mean values ± SD, *n* = 3 (biological replicates). Statistical analysis: Paired *t*‐tests.

Early exposure to FGF2, FGF4, FGF8, or FGF9 induces UCP1 expression in a resulting cell population that otherwise retains the identity of preadipocytes. We tested the hypothesis that fully differentiated adipocytes originating from these cells differ from adipocytes differentiated under control conditions. After FGF pre‐treatment, we induced differentiation using standard induction and differentiation cocktails (Figure 4A). After differentiation (i.e., 9 days since the end of FGF treatment), resulting adipocytes appeared morphologically indistinguishable upon visual inspection, FGF‐treated or not (Figure 4B). Likewise, fat accumulation into lipid droplets remained unchanged, both measured as total fat content and as lipid droplet number and size distribution (Figure 4C,D). In line, the thermogenic capacity of UCP1 in response to isoproterenol stimulation remained similar in control and FGF‐pretreated brown adipocytes (Figure 4E,F). Taken together, adipocytes differentiated from FGF‐treated preadipocytes are fully functional and morphologically unchanged compared to untreated cells. The FGF‐induced, UCP1‐positive preadipocyte population does not give rise to a different functional class of mature adipocyte.

### 3.5 Fibroblast Growth Factor Induced Preadipocyte UCP1 Expression Causes Lasting Changes to the Interferon Pathway in Mature Adipocytes

**FIGURE 5:**
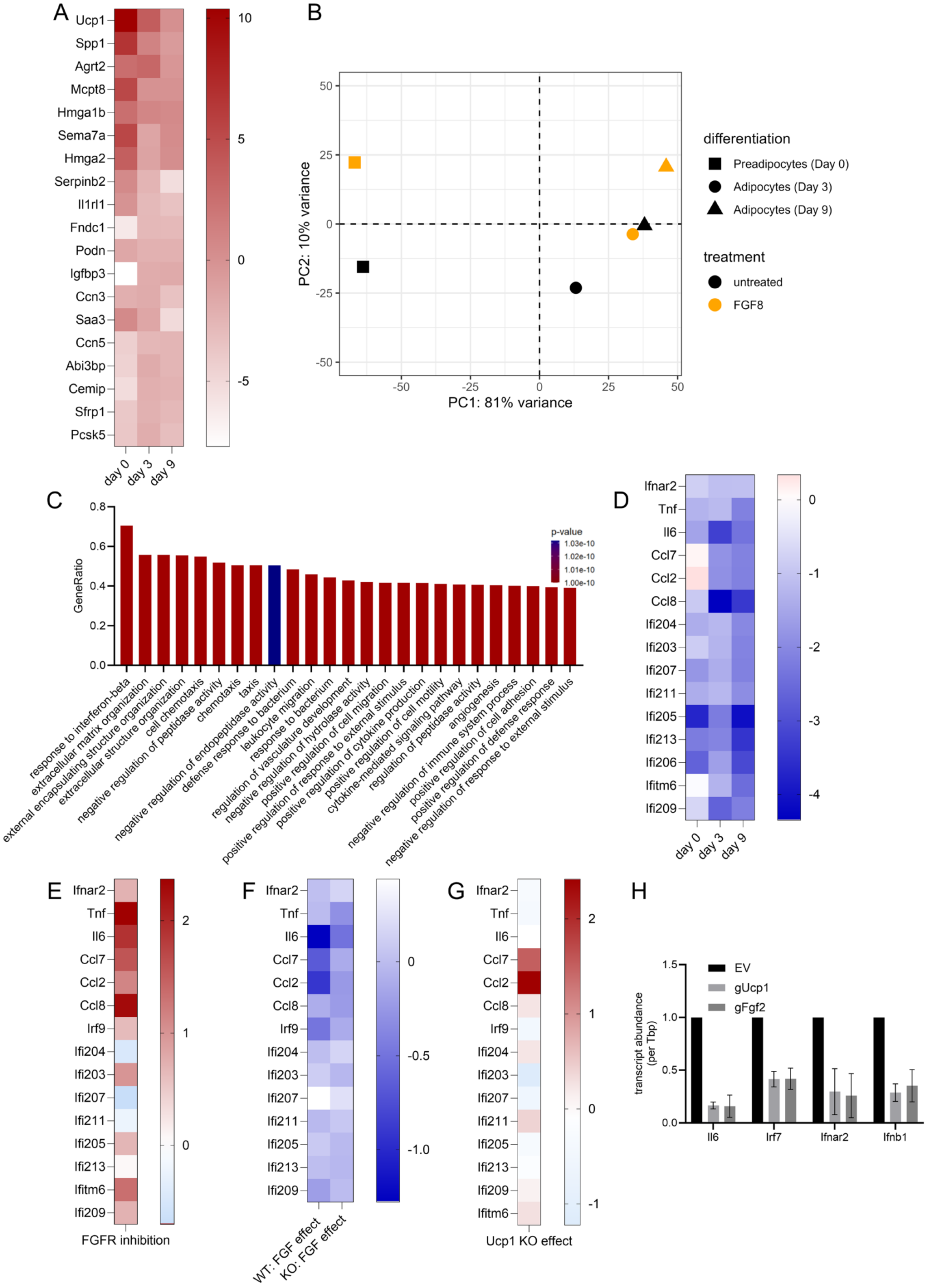
UCP1 mediates FGF‐induced suppression of inflammatory gene expression in brown adipocytes. (A) Heatmap showing fold changes in expression of Ucp1 signature genes—initially upregulated by FGF treatment in white preadipocytes—across brown preadipocytes, day 3, and day 9 stages, relative to untreated controls. (B) Principal component analysis (PCA) of transcriptomic profiles from brown preadipocytes pretreated with FGF8, followed by differentiation and bulk RNA sequencing at early (day 3) and late (day 9) stages. PCA was performed on variance‐stabilized transformed counts. (C) Gene Ontology (GO) term enrichment analysis of differentially expressed genes reveals suppression of cytokine‐mediated signaling pathways in FGF8‐pretreated adipocytes. (D) Heatmap of selected inflammation‐associated transcripts across brown preadipocyte, day 3, and day 9 time points, displaying fold change relative to untreated controls. Inflammatory gene expression remains broadly reduced following FGF8 pretreatment. (E) Heatmap showing fold changes in expression of inflammatory genes in wild‐type brown adipocytes pretreated with or without a transient FGFR inhibitor, followed by differentiation. (F) Heatmap showing fold changes in expression of inflammatory genes following FGF pretreatment in wild‐type (WT) versus Ucp1 knockout (KO) brown adipocytes. Cells were pretreated with FGF2, followed by differentiation. (G) Heatmap showing relative expression levels of inflammatory genes in interscapular brown adipose tissue (BAT) from 20‐day postnatal WT and Ucp1 knockout (KO) mice. (H) Overexpression of Ucp1 or Fgf2 via CRISPRa in brown preadipocytes suppresses IL6, Irf7, Ifnar2, and Ifnb1 expression after differentiation, showing that Ucp1 alone is sufficient to mediate the anti‐inflammatory response. Data represent mean ± SD; *n* = 3. Statistical analysis to compare FGF‐treated cells: One‐way ANOVA with Dunnett's test. *p < 0.05.

Mature adipocytes differentiated from FGF‐treated, UCP1‐positive brown preadipocytes appeared morphologically and functionally unaltered. We conducted a global transcriptome analysis of FGF‐treated preadipocytes and adipocytes at day 3 and 9 of differentiation (counting days after cessation of FGF treatment, that is, 2 days induction medium plus 1 or 7 days on differentiation medium) to detect other consequences of FGF pretreatment occurring acutely in preadipocytes (day 0), in non‐differentiated, non‐proliferating cells (day 3), and finally in fully differentiated, mature adipocytes (day 9). After acute treatment, we again observed UCP1 induction and the associated expression signature in preadipocytes, consistent with previous findings (Figure 5A). Over the course of differentiation, UCP1 induction gradually diminished to the level of untreated adipocytes. On day 9 of differentiation, none of the acutely upregulated transcripts remained different from controls. However, a principal component analysis (PCA) of non‐treated and FGF pre‐treated preadipocytes and adipocytes revealed a distinct and persistent separation of pre‐treated and non‐treated cells at all three differentiation states, proving a lasting effect of FGF treatment on the transcriptional profile (Figure 5B). This signature was dominated by down‐, not up‐, regulated transcripts. We applied a pathway analysis to identify the underlying pattern and detected significant attenuation of several pathways associated with interferon and immune response signaling (Figure 5C). Specifically, a large set of well‐known beta interferon downstream targets remained consistently and strongly reduced across all differentiation states, among them the key players of adipose tissue inflammation, interleukin‐6 (IL6) and tumor necrosis factor (Tnf) (Figure 5D).

We earlier observed FGF2 expression in preadipocytes in the absence of any exogenous FGF treatment (Figure 3H,J). We hypothesized that this endogenous FGF crosstalk will likewise serve the pre‐programming of an adequate inflammatory state of resulting adipocytes. Indeed, inhibition of endogenous crosstalk by a pan‐FGF receptor inhibitor during 2 days pre‐induction largely increased the inflammatory signature in fully differentiated adipocytes 9 days later (Figure 5E).

The suppression of inflammatory gene expression in FGF‐pretreated adipocytes raised the question of whether the presence of UCP1 in preadipocytes functionally contributes to this state in mature adipocytes. In support of this notion, brown adipose tissue of adult UCP1‐KO mice has been reported to feature an inflammatory phenotype including high levels of beta interferon, IL6, and TNF [27]. We thus compared the ability of preadipocyte FGF2 treatment to decrease adipocyte expression of the identified signature in cells isolated from wildtype and UCP1‐KO mice. In the absence of UCP1, early FGF2 treatment led to an impaired reduction in the identified inflammatory signature, suggesting a reduced FGF2 efficacy (Fig. 5F). The FGF‐mediated induction of UCP1 expression in preadipocytes was required for efficient programming of adipocyte inflammatory state.

FGF2 is already present in developing adipose tissue before birth (Figure 1H). We therefore chose to investigate the in vivo consequence of UCP1‐KO on BAT inflammatory gene expression in young mice (post‐natal day 21). Compared to wildtype BAT, about one half of the inflammation‐associated transcripts selected earlier proved increased in abundance, in the case of CCL2 and 7 strongly so (Figure 5G). The factors TNF and IL6, however, were not among the transcripts affected by UCP1 ablation, possibly due to their position downstream within the beta interferon signaling cascade.

The putative requirement of early UCP1 in preadipocytes for later proper control of adipocyte inflammatory gene expression prompted us to explore a permissive role of UCP1. We utilized a model of CRISPRa‐mediated transactivation of FGF2 or UCP1 in preadipocytes. Overexpression of FGF2 in preadipocytes leads to FGF2 release and paracrine/autocrine signaling mimicking exogenous FGF2 treatment, including the induction of UCP1, as demonstrated earlier [6]. Intriguingly, forced expression of UCP1 alone in preadipocytes led to a similar downregulation of IL6 and other candidates after full differentiation compared to forced FGF2 expression (Figure 5F). All of these were strongly reduced in mature adipocyte transcript abundance by both early FGF2 and early UCP1 expression in preadipocytes. Thus, UCP1 proved partially necessary and sufficient for FGF2‐driven programming of transcriptional activity of IL6 and other inflammatory genes, linking the presence of UCP1 in preadipocytes to the inflammatory status of mature adipocytes.

## 4 Discussion

Fibroblast growth factors (FGFs) are a diverse family of proteins involved in a plethora of developmental and metabolic processes. The subset of paracrine FGFs is characterized by the presence of a heparin‐binding domain that both limits mobility in the extracellular space and serves as a stabilizer during receptor interaction [11]. We and others previously reported the ability of several paracrine FGFs to induce the unusual presence of UCP1 protein in preadipocytes, prior to the initiation of adipogenic differentiation [6, 7, 8]. In this study, we systematically compared all paracrine FGFs for this ability and identified four FGFs—specifically FGF2, FGF4, FGF8, and FGF9—to induce UCP1 expression in both murine iBAT and iWAT preadipocytes. Among these, FGF2 elicited the strongest and most rapid UCP1 induction, was the only FGF endogenously present in appreciable amounts in preadipocytes and developing adipose tissue, and was secondarily induced by the other three candidate FGFs. These arguments render FGF2 the most likely physiological FGF acting on preadipocytes in vivo, in line with its capacity to trigger especially sustained FGFR signaling [28].

In principle, we found UCP1 protein to be functional when activated by the directly interacting activator TTNPB. The well‐established canonical signaling cascade activating UCP1‐mediated thermogenesis in brown adipocytes, however, is unlikely to be functional in preadipocytes lacking lipid droplets and adrenergic receptors. Furthermore, FGF‐induced UCP1 expression in preadipocytes was not accompanied by the well‐established gene expression signature enabling thermogenesis in brown and beige adipocytes. Indeed, FGF2/4/8/9 treatment did not give rise to an entirely novel cell population despite the untypical presence of UCP1. Instead, UCP1‐positive cells retained their preadipocyte identity and joined a previously rare, but pre‐existing preadipocyte subpopulation. Under control conditions, this population is likely the product of endogenous, paracrine/autocrine FGF2 signaling, as detected specifically among the preadipocytes of the stromal‐vascular fraction.

Full differentiation of FGF‐treated, UCP1‐positive preadipocytes did not lead to increased UCP1 abundance or activity in resulting mature brown adipocytes, nor did it induce morphological or adipogenic changes. It did, however, manifest in a strongly reduced inflammatory gene expression signature, specifically of targets of beta interferon, including the hallmark cytokine of adipose tissue inflammation, IL6 [29]. Beta interferon has been described as the master regulator of adipose tissue inflammation [30]. Both beta interferon and IL6 serve as vital hubs of mitogenic and thermogenic control in adipocytes [31, 32]. Notably, these changes were detected in mature adipocytes, 9 days after cessation of FGF treatment and after full adipogenic differentiation, demonstrating a highly persistent imprint on transcriptional control. This phenomenon also occurred endogenously in a crosstalk of primary preadipocytes, as demonstrated by an opposing imprint caused by FGF receptor inhibition.

Intriguingly, the initial finding of atypical UCP1 expression proved mechanistically linked to reprogrammed IL6 and other inflammatory markers in resulting adipocytes. In BAT of adult UCP1‐KO mice, IL6 is strongly increased together with many other transcripts of inflammation‐associated genes [27]. Surprisingly, even the presence of UCP1 in preadipocytes alone was sufficient to bring about changes similar to those in the adipocytes as FGF2 pretreatment or expression. At least part of the FGF2‐programmed changes in preadipocytes, which finally lead to an altered inflammatory state in the resulting adipocytes, is mediated through UCP1 in a necessary and sufficient manner.

The function of UCP1 in preadipocytes has been enigmatic since its first discovery. In the absence of adrenergic receptors and of significant lipid stores to mobilize, a classical thermogenic function through activation by free fatty acids appears unlikely. On the other hand, UCP1 is per se functional in this setting and uncouples respiration when activated. To be an active component in any mechanism, we must assume that activators are different from free fatty acids and have an uncoupling function that does not primarily serve thermogenesis. In one such scenario, proposed earlier and supported by multiple reports, UCP1 may serve to prevent excessive inner membrane potential and/or respiratory chain redox pressure and thereby defend against reactive oxygen species [33, 34, 35, 36]. The latter and their products have been reported to activate UCP1 [33, 37, 38]. Similarly, redox‐dependent UCP1 protein modification has been observed to regulate its activity [39] and, intriguingly, also inflammatory processes [40]. Plausibly, such non‐thermogenic functions of UCP1 are less or non‐important in brown or beige adipocytes, but a key feature in UCP1‐positive preadipocytes, a conjecture that remains to be tested.

From a translational perspective, our findings suggest that transient FGF2 signaling at the preadipocyte stage can durably reshape adipose immune characteristics, independent of classical thermogenic outcomes. Because chronic adipose inflammation is a central driver of insulin resistance and metabolic disease, such modulation may represent a strategy to improve systemic health [41]. Future in vivo studies will be required to determine whether short‐term modulation of FGF–UCP1 signaling can be targeted to prevent or reverse obesity‐associated inflammation.

Collectively, our findings demonstrate paracrine FGF2 signaling between preadipocytes to induce non‐canonical UCP1 protein expression. By an unresolved UCP1‐dependent mechanism, transcriptional regulation of inflammation‐related genes is negatively programmed to finally give rise to mature adipocytes with decreased inflammatory status, IL6, and beta interferon associated gene products. UCP1‐positive preadipocytes thus constitute a novel model to study endogenous, non‐thermogenic UCP1 protein function and its role in shaping adipocyte immune tone. Elucidating this process and its molecular underpinnings will provide potential targets for the manipulation of adipose tissue inflammatory status.

## Author Contributions

**Katharina Kuellmer:** conceptualization, investigation, formal analysis, writing – original draft, writing – review and editing. **Carolin Mönch:** investigation. **Wilma Roch:** investigation. **Christine Wurmser:** methodology, resources. **Patricia S. S. Petersen:** methodology, resources. **Hildigunnur Hermannsdóttir:** investigation. **Mersiha Hasic:** investigation. **Brice Emanuelli:** supervision, writing – review and editing. **Tobias Fromme:** conceptualization, supervision, funding acquisition, formal analysis, writing – review and editing.

## Conflicts of Interest

The authors declare no conflicts of interest.

## Data Availability

All transcriptomic datasets are available in the NIH Gene Expression Omnibus (GEO) database (accession numbers: GSE306159, GSE306209, GSE306412, GSE306413, GSE306079).
